# Supplementary material for: Hypertension screening, prevalence, treatment, and control at a large private hospital in Kampala, Uganda: A retrospective analysis
Source: PLOS Glob Public Health. 2022 May 10;2(5):e0000386. doi: 10.1371/journal.pgph.0000386 (PMC10021338; doi:10.1371/journal.pgph.0000386)
Supplement: S1 Table — (DOCX) [file pgph.0000386.s001.docx]

| Pharmacy Item Label | category |
| --- | --- |
| ALFUZOSINE 10MG*** | Peripheral Alpha-adrenoreceptor antagonists |
| AMLODIPINE 10MG | CCB |
| AMLODIPINE 5MG | CCB |
| AMLOZAAR-H | CCB |
| ATENOLOL 100MG | Beta Blockers |
| ATENOLOL 50MG | Beta Blockers |
| BENDROFLUAZIDE 5MG | Thiazide Diuretic |
| BISOPROLOL 2.5MG | Beta Blockers |
| BISOPROLOL 5MG | Beta Blockers |
| CAPTOPRIL 25MG | ACE Inhibitor |
| CARVEDILOL 12.5MG | Alpha + Beta Blockers |
| CARVEDILOL 6.25MG | Alpha + Beta Blockers |
| CLONIDINE 10MCG | Central Alpha-adrenoceptor agonist |
| DOXAZOCIN 4MG | Peripheral Alpha-adrenoreceptor antagonists |
| ENALAPRIL 10MG | ACE Inhibitor |
| ENALAPRIL 5MG | ACE Inhibitor |
| FRUSEMIDE 10MG/ML 2ML | Loop Diuretic |
| FRUSEMIDE 40MG | Loop Diuretic |
| HYDRALAZINE 20MG*** | Arteriolar smooth muscle relaxants |
| HYDRALAZINE 25MG | Arteriolar smooth muscle relaxants |
| INDAPAMIDE 1.5MG | Thiazide Diuretic |
| LABETALOL 200MG | Alpha + Beta Blockers |
| LABETOLOL 5MG/ML | Alpha + Beta Blockers |
| LISINOPRIL 10MG | ARB |
| LISINOPRIL 5MG | ACE Inhibitor |
| LOSARTAN 50MG | ARB |
| LOSARTAN 50MG+ HYDROCHLOROTH 12.5MG | ARB + Thiazide Diuretic |
| LOSARTAN POTASSIUM + AMLODIPINE 50MG + 5MG | ARB + CCB |
| METOLAZONE 5MG | Thiazide Diuretic |
| METOPROLOL 50MG | Beta Blockers |
| NEBIVOLOL 5MG | Beta Blockers |
| NEVIBILOL 5MG | Beta Blockers |
| NIFEDIPINE 10MG | CCB |
| NIFEDIPINE R 20MG | CCB |
| OLMESARTAN 40MG (CONSGT) | ARB |
| PRAZOSIN 1MG HYPOVASE 1MG*** | Peripheral Alpha-adrenoreceptor antagonists |
| PROPRANOLOL 40MG | Beta Blockers |
| S-AMLODIPINE 5MG | CCB |
| SPIRONOLACTONE 100MG | K- sparing Diuretic |
| SPIRONOLACTONE 25MG | K- sparing Diuretic |
| TELMISARTAN + HYDROCHLOROTHIAZIDE 80/12.5MG CO-MIC | ARB + Thiazide Diuretic |
| TELMISARTAN 40MG | ARB |
| TELMISARTAN 40MG AMLODIPINE 5MG (ARBITEL-AM) | ARB + CCB |
| TELMISARTAN 80MG | ARB |
| TELMISARTAN H 80/12.5MG | ARB + Thiazide Diuretic |
| TORSEMIDE 10MG | Loop Diuretic |
| VALSARTAN H 92.5MG (CONSGT) | ARB + Thiazide Diuretic |
| VERAPAMIL 80MG*** | CCB |
