# Supplementary figures and images for: Hypertension screening, prevalence, treatment, and control at a large private hospital in Kampala, Uganda: A retrospective analysis
Source: PLOS Glob Public Health. 2022 May 10;2(5):e0000386. doi: 10.1371/journal.pgph.0000386 (PMC10021338; doi:10.1371/journal.pgph.0000386)

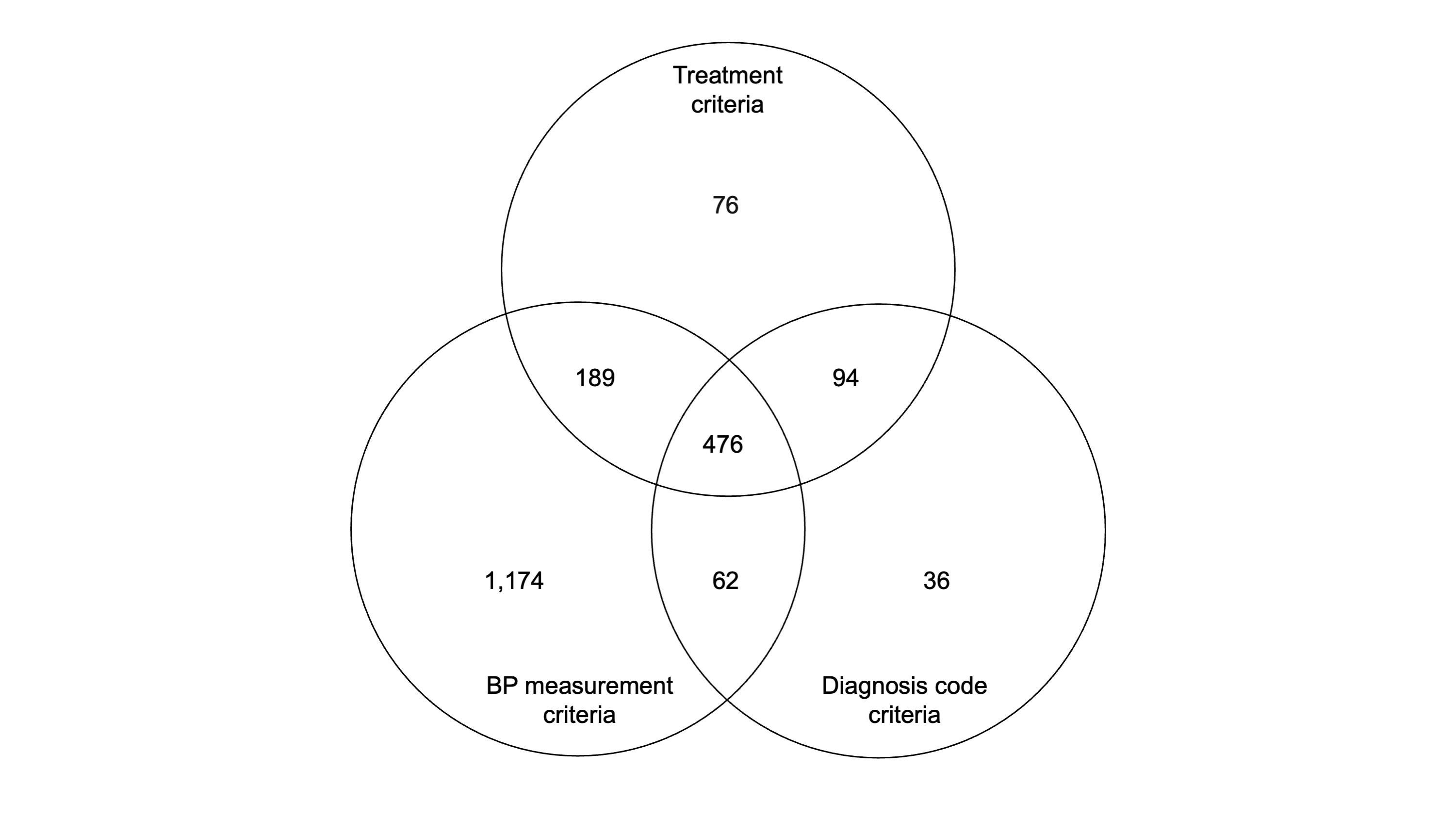

Supplement: S1 Fig — (TIFF) [file pgph.0000386.s002.tiff]
