# Supplementary material for: Hypertension screening, prevalence, treatment, and control at a large private hospital in Kampala, Uganda: A retrospective analysis
Source: PLOS Glob Public Health. 2022 May 10;2(5):e0000386. doi: 10.1371/journal.pgph.0000386 (PMC10021338; doi:10.1371/journal.pgph.0000386)
Supplement: Attachment — Submitted filename: PLoS GPH Reviewer Response 6-Mar-22.docx [file pgph.0000386.s003.docx]

PLoS Global Public Health

6 March 2022

Dear editors,

We thank the reviewers for their thoughtful comments regarding our recently submitted manuscript, “Hypertension screening, diagnosis, treatment, and control at a large private hospital in Kampala, Uganda: a retrospective analysis.” (PGPH-D-21-00519).

We have responded as requested to both reviewers’ comments, as well as the editor’s. Attached here are two versions of the manuscript: one with all revisions tracked, and the other with all revisions incorporated. Separately, we detail here exactly how and why we undertook these revisions, and where in the document each change may be found. Thanks to the reviewers’ astute observations, we have also noted and corrected several minor errors in our analysis, detailed below. None of these corrections to our quantitative results impact the overarching findings of our paper, or our discussion of same.

Thank you in advance for your further review of our submission. Please contact us with any further questions.

Best regards,

David Heller MD MPH

**Journal Requirements:**

1. *Please provide separate figure files in .tif or .eps format only.*

We have adjusted the document to remove the figure files and have reattached each in this format. Please advise if any further adjustments are necessary.

2. *Please update the completed 'Competing Interests' statement, including any COIs declared by your co-authors. If you have no competing interests to declare, please state "The authors have declared that no competing interests exist". Otherwise please declare all competing interests beginning with the statement "I have read the journal's policy and the authors of this manuscript have the following competing interests:*

We have revised the Competing Interests statement to refer to all COI expressly. We have only one item to report: Dr. Heller declares prior support from Teva Pharmaceutical Industries, but this entity did not sponsor, or interact with, any aspect of the current research.

3. *In the online submission form, you indicated that your data will be submitted to a repository upon acceptance.  We strongly recommend all authors deposit their data before acceptance, as the process can be lengthy and hold up publication timelines.*

We thank the editors for this request. We have uploaded within our revision files, with the permission of the hospital under study and in compliance with all local privacy laws, a de-identified dataset comprising all information used in our analyses, allowing for independent reproduction of our findings.

4. *Please amend your Financial Disclosure statement.*

We have revised this statement to reflect all funding for this work, inclusive of potential conflicts of interest such as the above.

**Editor Comments:**

*Please pay extra attention to the major issues raised by the reviewers. In addition to their comments, consider improving the discussion of the study limitations, particularly considering its design, setting, population and external validity.*

We thank both reviewers for their comments – in particular, Reviewer 2’s detailed examination of our analytic methodology. We feel the subsequent revisions have strengthened the paper considerably. However, we have separately revised the limitations section of the Discussion to underscore the unique context where the study took place – which limits its external validity – as well as its cross-sectional design, which impedes causal inference. See the final two paragraphs of the Discussion section for further details.

**Reviewer 1 Comments:**

*The limited data available for analysis – blood pressure values, visit characteristics (doctor type, insurance status), patient characteristics (age, sex) and items procured at the hospital pharmacy limit a more comprehensive comparison with public sector patients…It is this information that would be most useful for in-hospital quality audit of services rendered to optimize patient care and to allow for more informative comparison with hypertension care in public facilities for practice and policy.*

*The paper would significantly benefit from a more detailed comparison of hypertension care with previous studies undertaken in the country and elsewhere in the region, respectively sub-Saharan Africa.*

We thank the reviewer for noting this limitation of our study. We have added content to the Discussion section both comparing our work to other similar research in sub-Saharan Africa and acknowledging how our data (unusually, from a private sector hospital) differs from such work. In particular, as separately noted by the editor above, we have extensively revised the limitations section to acknowledge how our uncommon study approach limits external validity relative to other work in somewhat different settings.

**Reviewer 2 Comments:**

*The importance of the study subject in the study setting cannot be overemphasized. Hypertension remains one of the most important determinants of death and disability worldwide, with its impact increasing in LMIC, particularly in sub-Saharan Africa, and it is difficult to accept and worth studying why all we know so far is not translating into population benefit. I congratulate the authors for addressing this issue, making use of secondary data, namely routine care data, to do this study. However, I have some serious concerns regarding bias of several types.*

We thank the reviewer for her positive feedback regarding the subject matter of this paper, and our use of secondary data to address the problem of hypertension in a low-income country setting. We acknowledge, as detailed extensively below, the numerous methodological concerns the reviewer raises, which we have addressed as listed to the benefit of the manuscript.

1. *It is strange to have a population approach and a descriptive objective regarding prevalence of hypertension in the setting of one hospital. The authors should at least describe the system better, explain what they mean by primary care at hospital, and describe the population the hospital serves, for what purposes, how the patients get there (referral), etc. [title, line 297, and more]*

We acknowledge that this study approach is unusual in its use of only one hospital for a population-level descriptive approach – due to this hospital being both extremely high-volume and among the only private-sector hospitals of any size in the region. To justify our approach, and also to explain this unique context and its limitations more fully, we have added details regarding the study population and hospital mission and aims, as well as how patients access it, in line 101-106 of the Methods section. We kept the title unchanged for brevity.

2. *Line 32 – the word “efficacy” is not applicable here. Effectiveness, at best.*

We concur that the word “efficacy” implies misleadingly an intrinsic aspect of the care intervention rather than the outcomes it achieved in practice in this particular setting. Therefore, we revised to “effectiveness” in line 35 accordingly.

3. *Line 37 – two or more blood pressure checks regardless of the time interval between them? This could result in someone with only 2 blood pressure measurements separated by almost 1 year being misclassified according to the JNC principles. I suggest considering only 2 consecutive visits separated by no more than, say, 1 or 2 months.*

We thank the reviewer for this comment, and agree that consecutive blood pressure measurements of this frequency best align with JNC guidelines. However, given that blood pressure checks do not always occur at this frequency in Uganda, or even in high-income settings (see for example background references in Fiscella et al, *American Heart Journal*, 2015), we have elected to preserve a hypertension definition that includes consecutive visits further apart than two months, to capture as many potential cases of hypertension as possible. However, to investigate this concern, we calculated the median time between consecutive visits in the data set (29 days), with 25^th^ percentile duration of 6 days and 75^th^ percentile duration of 91 days. See these data added to the Results section, line 254.We however acknowledge, in the limitations section (line 529), that electing not to exclude patients with more than 2 months between consecutive visits might over- or under-estimate hypertension prevalence.

4. *Lines 38-39 – “an average of two blood pressures at separate…” should read “an average of two blood pressure measurements at separate…”*

We have made this correction to the abstract, as well as several others below, which we believe make the details of our abstract and methods section clearer and more precise.

5. *Line 41 – what is the coding system/classification used?*

We agree that the abstract is ambiguous regarding how we coded these data. We have edited the Methods section (see approximately line 195), to indicate expressly all the search codes (e.g., “hypertension” and “htn”- that we included, and those we excluded (e.g., “prehtn”) to detail this system in reproducible clarity.

6. *Line 44 – age-standardised prevalence is a meaningless value unless the standard is known, for comparison purposes. I know it is in the text, but the abstract must be understandable alone.*

We have revised the abstract to make this point clear, indicating in the abstract itself - where we refer to the Ugandan population pyramid (one of the youngest of any nation) standardized relative to the world population as a whole.

7. *Line 94 – “the term “hypertension specialty” is not clear and it doesn’t refer to a specific concept. I suggest “specialty visits considered most likely to xxxx”. I leave to the authors completing this sentence.*

We thank the reviewer for noting this ambiguity. As also noted in our response to other critiques below – and further clarified elsewhere in the revised manuscript – the hospital under study has general internal medicine and family medicine clinics which provide the bulk of hypertension care, but also has specialty clinics in cardiology and nephrology as well as a specialty clinic focused only on hypertension and diabetes Of note, we actually included only these two primary care clinics in the analysis, because we were unable to accurately discern which specialty care visits corresponded to these three clinics. We apologize for the prior error, which we have corrected in revisions to the paragraph the reviewer cites (roughly line 115).

8. *Line 94 – When the authors write “We included only visits to the hospital’s general internal medicine clinic and cardiology” do they mean beyond primary care (as written in the previous sentence) or truly only those?*

As per reviewer’s point 7 above, we concur that the nature and types of “primary care” and “specialty” clinics in the manuscript are ambiguous. Our goal was to include only the types of clinics that might be expected to detect and manage hypertension, inclusive of both primary care (namely, one general internal medicine clinic and one family medicine clinic) and specialty (namely, three specialty clinics focusing on cardiology; nephrology; and hypertension plus diabetes. However, we excluded the latter specialty clinics for the reasons detailed immediately above. Furthermore, we excluded clinics unlikely to screen for or manage hypertension (e.g. psychiatry) as well as those managing blood pressure syndromes different than essential hypertension (e.g, obstetrics). We have revised the manuscript accordingly within this paragraph.

9. *Internal medicine and cardiology are the specialties where patients with hypertension are likely referred to due to their hypertension. This population is not appropriate at all to have a population approach to hypertension prevalence or screening. THIS IS A MAJOR POINT.*

We thank the reviewer for this point and acknowledge its significance. As per point 1 above, we have revised the manuscript’s framing to note that we focus on one hospital with a large population of patients, rather than a general population of persons living in a given region as a whole. This approach necessarily carries many limitations relative to whether those who seek care in that hospital accurately reflect the community as a whole – but conversely, offers insights into precisely how those who *do* seek care in that hospital setting (in this case, a private hospital network) differ from the general population when compared with data gathered from the latter. We feel that our manuscript more accurately constitutes an analysis of the private-care-seeking population rather than a “population approach” for all persons in Kampala, and have edited the introduction and discussion accordingly – see lines 101-106.

Regarding the separate matter of whether the internal medicine and cardiology clinics capture the population within this hospital at risk of essential hypertension: as above, we have clarified that “primary care” includes all general medical clinics in which hypertension screening and treatment occurs (specifically general internal medicine and family medicine) and “specialty care” includes specialized clinics engaging with hypertension (specifically, cardiology, nephrology, and the hypertension plus diabetes clinic), all of which we in fact excluded from this study for the reasons detailed above. See detailed revisions in the Methods section, lines 122-123.

10. *Lines 100 and 103 contradict themselves – is the hospital protocol to measure BP in the non-dominant arm or in either arm?*

We thank the reviewer for noting this contradiction. The hospital protocol is to check blood pressure in either arm. We have corrected the discrepancy – see line 127.

11. *Line 110 – “when documented we also included diagnosis codes”. It is very important to describe how many visits (what proportion of visits) have at least one code documented.*

We agree this point is crucial to helping the reader understand how much of the data was usable for this study method. We have expressly noted in approximately line 135 that only 15.8% of of visits overall, and 13.2% of visits in persons with hypertension (as defined by our study criteria) lacked diagnosis codes. However, we did not exclude any visits in which diagnosis codes were absent.

12. *CONSORT is for randomized controlled trials, not applicable to this study.*

We have revised this section of the manuscript to avoid using the term or concept of CONSORT, given that the patient flowchart depicted here refers to a clinical care delivery model under study, rather than a randomized trial of an intervention.

13. *Line 121 and general question in the article – the outcome that the authors call “hypertension diagnosis” is in fact “hypertension prevalence”, defined as fulfilling criteria for diagnosis, which are then specified. Many of those patients were not exactly diagnosed. THIS IS A MAJOR POINT. The whole text, tables and figures need to be revised accordingly. In line 318 the authors themselves correctly say “persons meeting diagnostic criteria for hypertension”.*

We thank the reviewer for noting this important distinction. Given that a key purpose of our study was to ascertain what percentage of hypertension cases (i.e., hypertension prevalence) were in fact diagnosed (or undiagnosed) at the hospital under study, referring to all apparent cases of hypertension as “diagnoses” blurs a major distinction we are trying to examine.

We separately acknowledge that “diagnosis” implies meeting a set of clinical guidelines (i.e., “diagnostic criteria,” as above) – whereas the definition we used for cases of apparent hypertension differs slightly from most diagnostic guidelines for reasons related to limitations of our data set (see reviewer’s point 3 above). For both of these reasons, we revised the manuscript throughout to refer to “prevalence” of hypertension, per our own research criteria, rather than “diagnosis.’

14. *Lines 129-130 – it would be very important and useful to describe the system regarding access to medicines, supplies, distribution and patterns of use. Where else can patients get their medications? Are medical prescriptions necessary? How many patients who get one or more drugs at the hospital pharmacy can also get others during the year at other selling sites? THIS IS A MAJOR POINT.*

The reviewer’s point is well-taken. Briefly: patients at this hospital can and do access medications from other sources, including several local private pharmacies. However, medical prescriptions are necessary to obtain medications at these pharmacies.

Patients can, and likely do, obtain medications both at the hospital pharmacy and at other pharmacies – and unfortunately there is no reliable way to ascertain how frequently this outcome occurs. Anecdotally, we understand this outcome is uncommon due to the associated inconvenience – hence any medications at the hospital pharmacy would likely receive antihypertensive medications (if any) from the hospital pharmacy. Therefore, we structured our analysis to presume all (and only) persons who received other medications from the hospital pharmacy were eligible to get antihypertensives from this source. We have revised both the methods section and the limitations section (see in and around lines 176 and 534, among others) to clarify this point and acknowledge potential bias associated with this assumption.

15. *Line 131 – the most recent blood pressure value could be the second value that was considered for hypertension definition, in patients with only 2 visits or 2 BP measurements. This is unacceptable to define control. I suggest control should be considered only among treated patients and based on BP measurement AFTER treatment. Also, if this BP measurement is made too soon after treatment initiation (due to the study stop at 1 year) it may be inadequate to define control when “stable”, which takes several weeks.*

We thank the reviewer for this thoughtful point – we acknowledge that blood pressure control in treated hypertensive persons requires follow-up several weeks or more after initiation of treatment; and ideally, multiple such visits to confirm the “controlled” blood pressure measurement is correct. However, we also note that some hypertensive persons achieve blood pressure control without medication, and in this hospital setting many patients receive lifestyle counseling rather than, or prior to, medication for hypertension. We therefore elected to preserve our current approach, in which we define blood pressure control among hypertensive persons via the most recent blood pressure measurement regardless of whether treatment with medication occurred, or when blood pressure was checked (typically approximately a month after the prior visit).

We are mindful that this approach includes some persons with only two blood pressure measurements, as the reviewer notes - but we hypothesize these cases are infrequent. To investigate this hypothesis, we separately calculated the percentage of patients with exactly two blood pressure measurements who met criteria for hypertension (i.e., had an elevated average blood pressure) yet had a normal second blood pressure visit. This result was only 3.7%, indicating that persons who did not undergo follow-up visits or treatment after diagnosis were unlikely to have achieved spurious “control” because their second blood pressure check was normal.

We do intend to evaluate the blood pressure control levels of persons with and without treatment – including those with only two visits over the year – because we intend to capture how often such persons failed to receive a third follow-up visit for treatment, and went uncontrolled; how often persons received such a visit, received no medication, but still achieved control; and other hypertension care outcomes among persons who did not receive medication as treatment. We acknowledge, again, that the accuracy of this approach is limited by our short timeframe, as noted in the limitations section.

16. *Line 138 – considering the inclusion criterion of having at least one drug of any kind dispensed from the hospital pharmacy in one year seems to me to be too restrictive; I suppose much of this population may not get any drug either at this hospital or at any other place in a 1-year period, even if having medical appointments. Therefore, I suggest that this criterion should be applied only to define treatment and control (for the reason presented by the authors, which I understand) but not for hypertension prevalence, to avoid reducing representativeness so much.*

We thank the reviewer for raising this issue, which reveals a key ambiguity. The reviewer is correct that, in measuring hypertension prevalence (as well as treatment and control), we included only those persons who obtain medication at the hospital’s pharmacy. We made this decision because a key outcome of our work was the proportion of persons with hypertension who received treatment with medication – and to accurately measure the numerator and denominator of this result, we were required to examine only persons who obtain medication at the hospital (i.e., those whose treatment we could detect). Of note, in doing so, we excluded only 268 persons with hypertension who do not use the hospital pharmacy, while including 5,090 who do. Also of note, the prevalence of hypertension was 41.2% in persons not using the hospital pharmacy, similar to the 42% prevalence we report in those who filled medications at the hospital. We have revised the section around line 185 to clarify both the fact of this restriction in the prevalence data and the reason for doing so.

17. *Lines 145-149 – What if in the EMR it was written “high blood pressure” or “hypertensive”? On the other hand, how did the authors disregard other forms of hypertension such as intracranial hypertension, portal hypertension, pneumothorax, etc?*

We thank the reviewer for raising this issue, which reveals a key ambiguity in how we frame our approach. We created a series of codes for words that encompass essential hypertension but exclude other types of hypertension. We have expressly stated in approximately line 195 that these terms are hypertension”, “hypertensive”, “htn”, excluding “pre-hypertension”, “prehypertension”, “pre-htn”, and “prehtn”. Separately, in order to address the reviewer’s concern above, we have expressly searched for the terms and diagnoses of portal hypertension, intracranial hypertension, and pulmonary hypertension, and can confirm that no such examples appeared in the dataset nor were included in the analysis.

18. *Lines 150-152 – if the medications at the pharmacy are, fortunately, coded, don’t describe the data extraction as not having to use free text searches, but simply describe it the positive way, as being selected from a list.*

We acknowledge that the above language is unclear. We reviewed all the medications listed in the data set, and two co-authors with medical expertise (Dr. Majumdar and Dr. Heller) reviewed this list to agree on which medications constituted anti-hypertensive agents, and created a list of these agents in order to classify those under hypertension treatment. We have adjusted the language around line 198 accordingly to refer to selection from a list rather than a free text search.

19. *Lines 162-163 – I know it is difficult to do it in any other way (having the drug and the indication), particularly if not even the diagnoses are registered. However, you should at least discuss what other indications could be at stake, and if their prevalence is high or low in this population. Also, loop diuretics are probably not used for hypertension but rather heart failure, isn’t this right?*

We acknowledge that the multiple indications for antihypertensive agents (e.g., the use of angiotensin receptor blockers for heart failure) are a limitation of our study, unfortunately not readily surmountable due to lack of detailed clinical notes on medication rationale. To compensate for this issue, we elected to conservatively include any medication for which antihypertensive treatment is a listed indication, including furosemide (see for example drugs.com listing for same).

20. *Line 174 – by estimating screening rates at the visit level, did the authors take into account the interval between visits? For example, in a follow up visit very shortly after a previous visit (for example the next day, for the same reason, unrelated to hypertension) it would be reasonable not to measure BP again.*

We thank the reviewer for pointing out the issue of visits too closely spaced – in addition to the separate inquiry above regarding visits separated by more than two months. In order to make analytic use of as much of the data as possible, we did not exclude follow-up visits that were unusually close or far away from the prior visit, as justified above. However, we separately measured (for the purposes of this response letter) the number and proportion of follow-up visits that were 48 hours or less away from the prior visit. As noted above, visits only one or two days apart were uncommon (median duration 29 days; 25^th^ percentile 6 days), suggesting that the inclusion of such visits did not greatly impact the overall results. We have noted around line 158 that we did not place upper or lower bounds on the duration of time between two visits to include in our analysis. As noted above, we cite this matter (line 525) as a limitation of our analysis as well.

*21. Line 193 – it would be informative and important to describe the reasons/diagnoses in the 39235 visits.*

We agree that this context will be helpful in indicating how frequently hypertension occurs relative to other disease priorities. We have examined these data, broadly, and found that more than half the visits were “general checkup” of which the leading diagnoses were (among others) diabetes, heart failure, angina, and hypertension. We have added this content to roughly line 252 of the manuscript.

*22. Line 195 – please describe the distribution of the number of visits per patient among the 5090 patients (minimum, maximum, median, percentiles 25 and 75, for example) and the interval between consecutive visits.*

We agree that these data are important to understanding how this hospital handles hypertension (e.g., in terms of how frequently persons with hypertension are followed up) and also to understanding the extent of limitations to the data detailed above, such as follow-up visits that are much closer or further apart from the initial visit than might be expected. We have calculated the result, added to this section (line 254) accordingly: the mean number of visits was 4.25; median 3; interquartile 2-5; and maximum 32.

*23. Line 200 – I don’t think the word “attrition” is the best choice here. It suggests losses to follow up in the study.*

We thank the reviewer for noting this problem. There are indeed losses to follow-up in the hypertension care cascade, but also persons who are removed from the process appropriately – for example because they screen negative for hypertension – and others who remain in care but are inadequately treated. Instead, we explicitly refer to “imperfect treatment and control,” in lieu of “attrition,” at roughly line 261 to clarify we refer to gaps in how the care cascade performs overall – for instance persons who receive antihypertensive medication and follow-up but fail to achieve control.

*24. Table 1 – the difference may not be statistically significant, strangely, but from 60% to 81% insured is a large and very important difference. Its impact should be discussed. Statistical significance is not important here because this comparison of characteristics is not hypothesis testing. It is the magnitude of the difference that matters.*

We agree this marked difference is worth noting, and acknowledge that statistical significance is not pertinent to its relevance. We cover this matter in the discussion section – noting for instance that the diagnosed cohort includes only those who are treated at the hospital pharmacy, in addition to other potential unmeasured differences in their socioeconomic status (e.g. income, education) that may correlate with hypertension status.

*25. Lines 216-217 – it is very strange to register only systolic or only diastolic BP in so many patients. How do the authors explain this, taking into account their knowledge of local practice?*

We are aware that this problem is surprising and unexpected. However, we regret that the listed numbers regarding its frequency are incorrect. In fact, only five blood pressure readings were excluded due to inclusion of systolic or diastolic results alone. We have corrected this typographic error, which does not affect the rest of our results, around line 282. Further, we have discussed the matter with two co-authors familiar with hospital operations and data capture (Dr. Gidio and Mr. Sentamu) who report that nurses and other hospital staff almost never fail to measure both systolic and diastolic blood pressure – in part because the automated cuffs always generate both numbers. This issue is therefore almost certainly a problem of data capture and information technology.

*26. Line 218 – The 12821 patients with “at least 1 blood pressure measurement during the study period” include those with only systolic or only diastolic?*

These data include only those with systolic and diastolic results, as we now state in this section. As above, we removed all visits in which only one of the two numbers was recorded. Of note, there were only five such visits in total, not the many previously documented.

*27. Line 221 – the association with number of visits is very likely to represent reverse causality. This limitation could be addressed by taking into account the reason for the visits.*

We agree that number of visits may associate with screening due to reverse causality among other factors. However, our dataset does not detail the reasons for each visit, precluding further evaluation of this hypothesis. We have noted this limitation, like others above, in the relevant section of the discussion.

*28. Table 2 – the limits of the age categories are above or below? For example: 18-30, 30-45 – in which class are those aged 30?*

We thank the reviewer for noting this error. Age categories terminate immediately after (above) the subsequent category. Therefore the first category above is 18-30, and the second is 31-45, and so on. We have corrected this Table 2 text accordingly.

*29. Lines 236-238 – I suggest drawing and showing a Venn diagram with the 3 criteria, to show how many patients were common among them.*

We have created a Venn diagram accordingly, as we agree that the substantive overlap among these three criteria may create confusion. See figure attached in our manuscript revision and referenced in the manuscript at approximately line 321.

*30. Line 244 – the logistic regression model in this study assesses association, not prediction.*

We agree this term is not correct, as causality (and hence prediction) is not discernable with this model. We have corrected the error at this line.

*31. Table 3 – the data shown are not trends. They are just numbers.*

We agree this term is inappropriate. We have changed the Table 3 title to “Prescription Data” instead.

*32. Table 3 – clonidine and alfuzosine are swapped (change categories)*

We are grateful the reviewer noted this typographic error. We have switched these medications to their correct types in Table 3.

*33. Line 272 – how can untreated patients be controlled? Probably the problem is in the diagnosis as a case… The hundreds of papers on hypertension prevalence, awareness, treatment and control, estimate control among the treated or control among all hypertensives (defined and treated and controlled). It is not seen as defined here.*

As discussed above, we define “treatment” in terms of blood pressure medication, though other forms of treatment, such as lifestyle change, are sometimes sufficient to control hypertension. We therefore measured control levels both in those treated (with medication) and untreated with medication (but perhaps treated by lifestyle change). We have edited this section, and the above sections defining treatment, to clarify that we refer only to pharmacotherapy as the treatment in question. Therefore, blood pressure control can and does occur in “untreated” persons by other therapeutic means, such as lifestyle change. We concede that some persons who achieved blood pressure “control” without medicine may have simply had normal blood pressure by chance at their most recent visit (see point 15 above) but, as estimated above, suggest that these cases are relatively uncommon.

*34. The association between treatment and control is weak (OR close to 2). Please discuss this more thoroughly*.

We agree this is a noteworthy point. As discussed above, the reasons for control among the untreated may include lifestyle changes or other non-pharmacologic intervention among those not “treated” with medication, as well as spurious results due to a single chance normal blood pressure as the final measurement in a person whose average blood pressure remains elevated. We discuss these and other explanations and limitations in more detail in this section of the discussion (see line 541).

*35. Lines 327-328 – inability to afford medication is repeated*

Apologies for the error. We have removed the duplication in this line.

*36. Lines 399-410 – this paragraph is too long for a conclusion. Please stick to the direct answer to the study objective, without discussing the results.*

We agree that this paragraph could be more succinct, and have focused on the implications of the findings for our overall study objective rather than the details of the results per se.

*37. Figure 2 – The fourth bar title should read “At least 2 BP measurements”. At least means it could be more. The third bar should disappear, in my opinion (see comment 16, above). The fifth bar title should read “Criteria for hypertension”.*

We agree that these corrections and revisions make Figure 2 clearer, and have edited the figure accordingly.

*38. Figure 3 – Were age and number of visits included as continuous variables in the models? The effect of the number of visits is probably non-linear, so this option might not be the best.*

We indeed treated both of these variables as continuous, but acknowledge that the number of visits is necessarily an integer and therefore could have also been approached categorically. We took this approach due to the wide range in number of visits (see point 22 above), and also because we were (and remain) unsure whether the effect of the number of visits is indeed non-linear. We note this decision as a potential limitation of our analytic approach in the limitations section of the discussion as well (line 555).
